# Cutaneous Metastases from Lung Adenocarcinoma

**DOI:** 10.1155/2020/8880604

**Published:** 2020-08-12

**Authors:** Yu Wang, Ruzeng Xue

**Affiliations:** ^1^Department of Dermatology, Dermatology Hospital, Southern Medical University, Guangzhou, China; ^2^Department of Dermatology, Guangdong Provincial Dermatology Hospital, Guangzhou, China

## Abstract

Cutaneous metastases are unusual presenting symptoms of lung cancer. Therefore, they are prone to be misdiagnosed and missed. The report describes a case of a forty-nine-year-old female with painful zosteriform rashes showing multiple vesicle-like papules localized on the left breast for 10 days. The patient had been diagnosed as lung adenocarcinoma at the department of oncology one year ago. Skin biopsy revealed blue nodular lesions in the dermis, composed of clustered heterogeneous tumor cells with glandular formation. Immunohistochemical stains confirmed the diagnosis of metastatic lung adenocarcinoma.

## 1. Introduction

Lung cancer can metastasize to almost all organs but more often invades the hilar nodes, liver, adrenal glands, bones, and brain [1]. The incidence of lung cancer with metastases to the skin varies between 1–12% [1]. A lung cancer metastasis is usually classified only as adenocarcinoma, squamous cell carcinoma (SCC), or undifferentiated carcinoma. Until the 1980s, SCC was reported as the most common type of lung cancer. However, adenocarcinoma has replaced SCC as the most common lung cancer subtype, especially in women and in never-smokers. Sun et al. reported that the type of adenocarcinoma was 3.4 times more frequent than that of SCC [2]. Skin metastases can appear on any cutaneous surface, and the most common sites are the chest, abdomen, head, and neck [3, 4]. Cutaneous metastases have various manifestations, such as single papules/nodules or multiple lesions on anywhere of the skin, while other rare forms may show plaque-like lesions, erysipelas-like papules, zosteriform lesions, and scars [3, 5].

## 2. Case Presentation

A forty-nine-year-old nonsmoker female was admitted to our department with multiple painful papules localized on the left breast. They appeared eruptively for about 10 days and initially diagnosed as herpes zoster in another hospital. The patient had been diagnosed as lung adenocarcinoma at the department of oncology one year ago. She was given oxitinib mesylate, a targeted drug for the treatment of non-small-cell lung cancer. In addition, the patient exhibited symptom of pain, signs of weight loss, anorexia, and fatigue.

**Figure 1:**
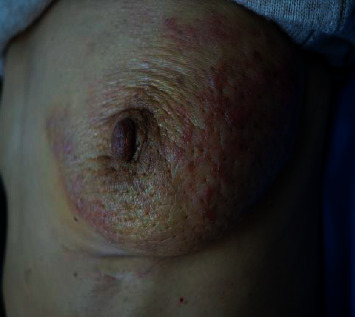
Zosteriform vesicle-like papules, measuring 0.5–1.0 cm on the left breast. Pink or red, firm, and tender.

**Figure 2:**
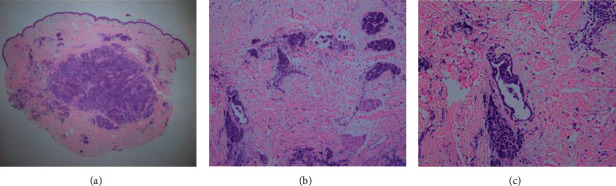
Skin biopsy revealed (a) blue nodular lesions infiltrating in the dermis, composed of clustered heterogeneous tumor cells with glandular formation (H&E, magnification: ×20); (b) some tumor cells were detected within vessels or lymphatic vessels (H&E, magnification: ×40); (c) some cells were blue and transparent, and mitosis was significant (H&E, magnification: ×200).

**Figure 3:**
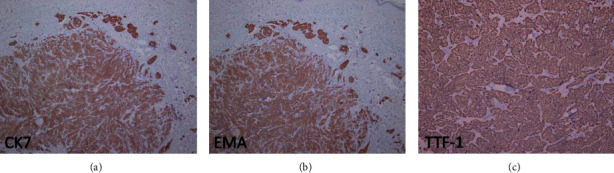
Immunohistochemical stain highlighting the tumor cells, showing (a) CK-7, (b) EMA, and (c) TTF-1 positive.

Physical examination showed zosteriform vesicle-like papules, measuring 0.5–1.0 cm on the left breast. The lesions were pink or red, firm, and tender (Figure 1). Excisional biopsy was performed revealing blue nodular lesions infiltrating in the dermis, composed of clustered heterogeneous tumor cells with glandular formation. Some tumor cells were detected within vessels or lymphatic vessels. Some cells were transparent. Mitosis was significant (Figures 2(a)–2(c)). In immunohistochemistry, tumor cells were positive for cytokeratin (CK), cytokeratin-7 (CK7), thyroid transcription factor-1 (TTF1), and EMA and negative for cytokeratin-20 (CK20), carcinoembryonic antigen (CEA), and gross cystic disease fluid protein-15 (GCDFP-15) (Figures 3(a)–3(c)). Proliferative index, as measured by Ki-67, was approximately 45–50% of tumor cells. According to the clinical and pathological features, cutaneous metastatic lung adenocarcinoma was made.

## 3. Discussion

Skin metastases suggest the progression of primary cancer and portend a poor clinical prognosis. Skin metastases from lung cancer are rare. The percentage of patients with lung cancer that develops cutaneous metastases ranges from 1 to 12 percent [1]. It is seen more often in men than in women [6]. It does not show any specific presentation. It is often painless and less likely to be noticed, making it more difficult to be diagnosed correctly, which may delay treatment. Although described cases show that metastatic nodules are painless, our patient showed severe pain. The presence of zosteriform painful vesicle-like lesions really mimics herpes zoster clinically in our case.

The mechanisms determining the metastasis of lung cancer in skin remain unknown. Pathogenesis is suggested to be by lymphovascular invasion, with poor differentiation and upper lobe tumors increasing the risk [7]. Usually, skin metastasis develops after initial diagnosis of the primary malignancy and late in the course of the disease. Occasionally, skin lesions that arise from lung cancer may develop before the primary tumor is recognized. In our case, skin metastases occurred during the immunotherapy. Histology shows most commonly adenocarcinoma and then squamous/small-cell followed by large-cell carcinoma [1]. Immunohistochemical markers are useful for the identification of the primary cancer or when a shorter differential is desired. Anti-TTF is both sensitive and specific for primary adenocarcinomas, bronchioalveolar carcinomas, and small-cell carcinomas when thyroid primary is excluded [8]. CK7+and CK20− are sensitive but not specific for primary adenocarcinomas and bronchioalveolar carcinomas. The CK7+/CK20−tumors usually include the lung, breast, endometrium, ovary, thyroid, salivary gland, and mesothelioma [8, 9].

Treatment of a single solitary skin lesion usually includes surgery alone or combined with chemotherapy, and/or radiation. If lesions are more disseminated, chemotherapy is the primary option but may elicit an inadequate response [10]. Radiation can also be used alone and/or in combination with chemotherapy, and/or surgery. However, despite the combination of radiotherapy and chemotherapy, patients with lung cancer developing cutaneous metastases have a poor outcome. Mean survival is short, usually 5 to 6 months after diagnosis of cutaneous metastasis [1].
